# Physical activity in adults with controlled and uncontrolled asthma as compared to healthy adults: a cross-sectional study

**DOI:** 10.1186/2045-7022-3-1

**Published:** 2013-01-15

**Authors:** Annelies Verlaet, Andre Moreira, Ana Sá-Sousa, Renata Barros, Rute Santos, Pedro Moreira, Joao Fonseca

**Affiliations:** 1 Faculty of Nutrition and Food Sciences, University of Porto, Rua Dr. Roberto Frias, 4200-465, Porto, Portugal; 2 Immunology, Faculty of Medicine, University of Porto, Al. Hernani Monteiro, 1, 4200-465, Porto, Portugal; 3 CINTESIS and Health Information and Decision Sciences, Faculty of Medicine, University of Porto, Porto, Portugal; 4 Research Centre in Physical Activity, Health and Leisure, Faculty of Sport, University of Porto, Porto, Portugal; 5 Maia Institute of Higher Education (CIDAF), Maia, Portugal

**Keywords:** Asthma, Bronchoconstriction, Asthma control, Physical activity, Exercise, International physical activity questionnaire

## Abstract

**Background:**

Though exercise-induced bronchoconstriction is common among asthmatics, physical activity (PA) seems important in asthma management. Still, various studies point at avoidance of sports and certain daily life activities like walking stairs, even by patients with mild symptoms. We aimed to compare physical activity levels between healthy subjects and asthmatics with controlled and uncontrolled disease.

**Methods:**

Data on asthma and PA were drawn from the Portuguese National Asthma Survey. The short telephone version of the International Physical Activity Questionnaire (IPAQ) was used to measure PA levels. Current asthma was defined as self-reported asthma and at least one of these criteria: one or more asthma symptoms in the last twelve months, currently taking asthma medication or an asthma medical appointment in the previous twelve months. Controlled asthma was defined as a CARAT global score > 24 or a CARAT second factor score ≤ 16. Healthy subjects were defined as individuals without atopy, heart disease or any respiratory symptom. X^2^ and Mann–Whitney/Kruskall-Wallis tests were used to compare groups. Logistic regression analyses were performed to assess relations between asthma status and PA dimensions.

**Results:**

A total of 606 non-asthmatics, 125 controlled and 78 uncontrolled asthmatic subjects were included. In both genders, overall PA level did not differ significantly between groups. Controlled (men) and uncontrolled (women) asthmatics did more vigorous PA than healthy respondents. Male controlled asthmatics also did more moderate PA. Crude logistic regression showed positive relations between daily sitting time, vigorous and moderate PA and controlled asthma in men and between vigorous PA and uncontrolled asthma in women. After adjustments for confounders, moderate PA remained a predictor of controlled asthma in men, while vigorous PA doubled the risk of uncontrolled asthma in women.

**Conclusion:**

Our study showed that adult asthmatics, independent of asthma control, do not seem to have a more sedentary lifestyle than their peers. Nevertheless, PA should be encouraged, as only about half of them reached activity recommendations.

## Background

The increase in the prevalence of asthma observed in most developed countries has been accompanied by important changes in lifestyle, like walking or cycling for transportation. Reduced physical activity has been associated with increased asthma prevalence [1], and increasing physical activity levels has been suggested to prevent disease progression [2]. Physical training may reduce breathlessness and asthma symptoms by strengthening respiratory muscles and by decreasing ventilation rate during exercise. Training programs in asthma have not, however, improved lung function in controlled trials.

Though many asthmatics suffer from exercise-induced bronchoconstriction (EIB), they might specifically benefit from adequate physical activity (PA) [2]. In addition, EIB can be well controlled by medication in most of them [3]. The vast majority of asthma patients should therefore try to achieve the recommended levels of at least 30 minutes, 5 days per week, or 20 minutes, 3 days per week, of moderate or vigorous aerobic physical activity, respectively [4].

Still, various studies point at avoidance of sports and certain daily life activities (e.g. walking stairs), even by patients with mild symptoms, and lower energy expenditures from leisure-time PA and more inactivity in asthma patients than in controls [5-7]. However, higher physical activity levels in asthmatics were also found [8], as well as no differences in overall exercise frequency or duration [9]. Data on activity levels in asthma are thus inconsistent and little is known about differences between asthmatics with controlled and uncontrolled disease. Therefore, this study aims to comparatively assess physical activity levels in adults with controlled and uncontrolled asthma and healthy individuals.

## Methods

Data collection was based on the Portuguese National Asthma Survey - *Inquérito Nacional sobre Asma* (INAsma), a nationwide population-based cross-sectional telephone interview survey that included two phases.

Sample size calculation, based on an asthma prevalence of 6% in the general population [10] and considering attrition rates of 20-40% and unstable variables (e.g. non-response), revealed that 665–776 asthmatics needed to be identified and 7,387-12,927 households to be interviewed.

The first phase of the INAsma aimed to estimate the prevalence of asthma symptoms in the Portuguese population. The study design was described in detail elsewhere [11]. Briefly, to obtain a representative sample of the general population, a stratified cluster sampling was used. We selected a random sample of households within each municipality (stratum), using a list of landline phone numbers. Within each selected household, the last household resident to have his/her birthday was selected. If this individual was younger than 15 years we interviewed the caregiver. Individuals unable to understand spoken Portuguese or with cognitive or physical conditions hampering the interview were excluded. The phone interview was based on the GA^2^LEN survey [12,13]. The simple response rate was 40%; the corrected response rate 50%.

The second phase mainly aimed at estimating the proportion of asthma patients with controlled disease in Portugal. Asthmatic and healthy respondents from the first phase were contacted again, as well as asthmatic household members from first phase respondents. PA was measured with the short telephone version of the International Physical Activity Questionnaire (IPAQ), using last week recall. Asthma control was assessed by CARAT, the control of allergic rhinitis and asthma test [14-16].

*Current asthma* was defined as self-reported asthma and at least one of these criteria: one or more symptoms (wheezing, waking up with breathlessness or having an asthma attack) in the last twelve months, currently taking asthma medication or an asthma medical appointment in the previous twelve months.

*Controlled asthma* was defined as a CARAT global score > 24 or a CARAT second factor score ≥ 16 [14-16].

*Healthy* subjects were defined as individuals without atopy, heart disease or any respiratory symptom related to asthma, bronchitis, rhinitis or sinusitis.

*Walking, moderate and vigorous PA,* as well as *total PA*, were expressed as MET-min/week (metabolic equivalent) [17]. For each PA level and *daily sitting time*, respondents were classified according to their gender-specific median value (≤ median and > median) [18], as well as according to the ACSM/AHA 2007 PA guidelines [4]. *Overall PA* level was classified in the health-enhancing, moderate or low level PA category [17]:

● Health-enhancing PA level (HEPA) was defined as a) vigorous activity on ≥ 3 days achieving ≥ 1500 MET-min/week, or b) ≥ 5 days of walking, moderate and/or vigorous activities achieving ≥ 3000 MET-min/week.

● *Moderate PA level* was defined as a) ≥ 3 days with ≥ 20 minutes of vigorous activity, b) ≥ 5 days with ≥ 30 minutes of moderate activity and/or walking or c) ≥ 5 days of walking, moderate and/or vigorous activities achieving ≥ 600 MET-min/week.

● *Low PA level* was defined as any activity level not meeting the criteria for moderate or health-enhancing PA.

● *Body mass index* (BMI) was calculated from self-reported weight and height (kg/m^2^). *Socioeconomic status* (SES) was categorized as high (A social class), medium (B and C social classes) and low (D social class), based on the occupation and education of the person who financially contributed most for the household. *Smokers* were defined as respondents having smoked at least one cigarette per day or one cigar per week during one year. *Current smokers* smoked in the last month, e*x-smokers* reported having quit smoking at least one month preceding the survey.

**Figure 1:**
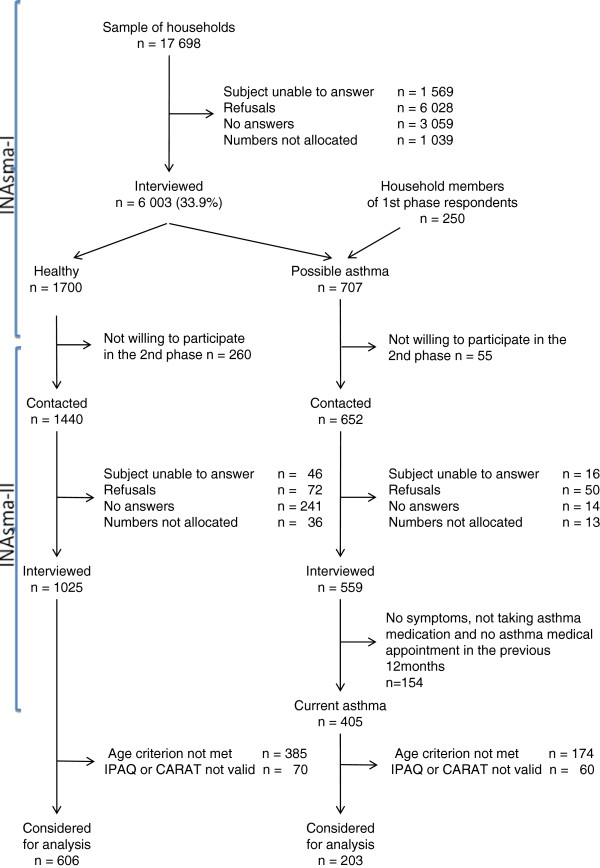
**Participants flowchart.** From the 17 698 households contacts, 6 003 participants were included in the 1^st^ phase and 1584 in the 2^nd^ phase of INAsma survey. A total of 606 healthy and 133 asthmatics were considered for physical activity analysis.

Data entry was done automatically by Computer Assisted Telephone Interviews (CATI). Only individuals who met the age criterion (18–69 years) and with valid IPAQ and CARAT answers were considered for analysis (Figure 1). Truncation rules were applied. Data were analyzed separately for men and women. Categorical variables are presented as frequencies and percentages, continuous variables as median and interquartile range (IQR). χ^2^ tests and Mann–Whitney/Kruskall-Wallis tests were used to compare groups. Crude and adjusted logistic regression analyses were performed to assess associations between asthma and PA. P-values < 0.05 were considered statistically significant. Adjustments were made for variables significantly associated with both asthma status and any PA level: age and BMI for men and age for women (data not shown). For the multinomial logistic regression, a main effects model was used. Results are presented as odds ratios (ORs) with the respective 95% confidence intervals (95%CI). Data were analyzed by using SPSS Statistics 17.0.

The study was approved by a Hospital Ethics Committee (*Comissão de Ética do Hospital de São João, Porto*). All participants gave oral informed consent and were informed that they could abandon the study whenever they pleased. Data confidentiality was guaranteed by storing personal information separately from the study data.

## Results

From the 809 participants, 606 were classified as non-asthmatic, 125 as controlled and 78 as uncontrolled asthmatic (Table 1). Half of the asthmatics and non-asthmatics reached the 2007 activity recommendation (54.6% and 48.5%, respectively). In both genders, overall PA level and total PA MET-minutes per week did not differ significantly between groups. Male controlled and female uncontrolled asthmatics did more vigorous PA than their corresponding healthy respondents (male, median(IQR): 960.00(2880) and 340.00(1920), respectively and women, median (IQR): 0.00(1380) and 0.00(560), respectively, but no significant differences were found when both genders were combined. Male controlled asthmatics also did more moderate PA and sat more than the healthy men (MPA, median (IQR): 280.00(960) and 60.00(720), respectively and sitting time, median (IQR): 300.00(330) and 240.00(240), respectively).

**Table 1:** Characteristics and physical activity of the participants by gender and asthma status

	**Men (n = 371)**			**Women (n = 438)**			**Total (n = 809)**		
	**Healthy (n = 300)**	**Controlled asthma (n = 59)**	**Uncontrolled asthma (n = 12)**	**Healthy (n = 306)**	**Controlled asthma (n = 66)**	**Uncontrolled asthma (n = 66)**	**Healthy (n = 606)**	**Controlled asthma (n = 125)**	**Uncontrolled asthma (n = 78)**
**Age, years , median (IQR)**	50.0 (28.0)*	36.0 (25.0)*	56.5 (15.0)*	55.00 (20)	50.00 (28)	53.50 (22)	53.0 (24)*	43.0 (28.0)*	54.0 (21.5)*
**BMI, median (IQR)**	25.4 (5.0)	24.7 (4.0)	27.6 (4.0)	24.67 (6)	24.98 (7)	26.26 (8)	25.1 (5)	24.9 (5.1)	26.6 (6.6)
**SES, n (%)**									
High	26 (8.8)	10 (16.9)	2 (16.7)	15 (5.0)	4 (6.1)	1 (1.5)	41 (6.8)	14 (11.2)	3 (3.9)*
Medium	238 (80.4)	45 (76.3)	7 (58.3)	224 (73.9)	56 (84.8)	49 (75.4)	462 (77.1)	101 (80.8)	56 (72.7)
Low	32 (10.8)	4 (6.8)	3 (25.0)	64 (21.1)	6 (9.1)	15 (23.1)	96 (16.0)	10 (8.0)	18 (23.4)
**Smoking, n (%)**									
Never	147 (49.0)	30 (50.8)	5 (41.7)	259 (84.6)	50 (75.8)	51 (77.3)	406 (67–0)	80 (64.0)	56 (71.8)
Ever	153 (51.0)	29 (49.2)	7 (58.3)	47 (15.4)	16 (24.2)	15 (22.7)	200 (33.0)	45 (36.0)	22 (28.2)
**Daily sitting time, min, median (IQR)**	240.0 (240.0)**^,§^	300.0 (330.0)**^, §^	180.0 (195.0)**	180.00 (180)	180.00 (180)	180.00 (210)	180.0 (180.0)**	240.0 (300.0)**	180.0 (187.5)**
**VPA, MET-min/week, median (IQR)**	**340.0 (1920.0)**^**§**^	**960.0 (2880.0)**^**§**^	960.0 (5130.0)	0.00 (560)**^, §§^	0.00 (960)**	0.00 (1380)**^, §§^	0.0 (960.0)	240.0 (1440.0)	0.0 (1920.0)
**MPA, MET-min/week, median (IQR)**	60.0 (720.0)^**§**^	280.0 (960.0)^**§**^	120.0 (720.0)	280.00 (3360)	280.00 (3360)	450.00 (3360)	280.0 (1680.0)	280.0 (1680.0)	360.0 (3360.0)
**WPA, MET-min/week, median (IQR)**	346.5 (693.0)	594.0 (941.0)	577.5 (1361.0)	429.00 (759)	346.50 (743)	346.50 (594)	396.0 (742.5)	462.0 (1089.0)	371.3 (594.0)
**Total PA, MET-min/week, median (IQR)**	1657.5 (3627.0)	2000.0 (4194.0)	2253.0 (6373.0)	1680.00 (4347)	1732.50 (5993)	2273.00 (4499)	16660. (3861.6)	1878.0 (5579.5)	2273.0 (4499.3)
**Overall PA level, n(%)**									
Low	91 (30.3)	18 (25.0)	3 (25.0)	83 (27.1)	22 (33.3)	17 (25.8)	174 (28.7)	40 (32.0)	20 (25.6)
Moderate	114 (38.0)	24 (40.7)	5 (41.7)	114 (37.3)	21 (31.8)	23 (34.8)	228 (37.6)	45 (36.0)	28 (35.9)
HEPA	95 (31.7)	17 (28.8)	4 (33.3)	109 (35.6)	23 (34.8)	29 (39.4)	204 (33.7)	40 (32.0)	30 (38.5)
**2007 PA recommendation, n(%)**									
No	161 (53.7)	27 (45.8)	5 (41.7)	151 (49.3)	31 (47.0)	29 (43.9)	312 (51.5)	58 (46.4)	34 (43.6)
Yes	139 (46.3)	32 (54.2)	7 (58.3)	155 (50.7)	35 (53.0)	37 (56.1)	294 (48.5)	67 (53.6)	44 (56.4)

*Comparison between healthy, controlled asthmatic and uncontrolled asthmatic, p=0.01. ** Comparison between healthy, controlled asthmatic and uncontrolled asthmatic, p<0.05. ^**§**^ Comparison between healthy men and controlled asthmatic men, p<0.05. ^**§§**^ Comparison between healthy and uncontrolled asthmatic women, p<0.05. BMI: body mass index; PA: physical activity; VPA: vigorous PA; MPA: moderate PA; WPA: walking PA; Total PA: walking, moderate and vigorous PA; HEPA: health-enhancing PA.

Crude logistic regression showed positive associations between daily sitting time, vigorous and moderate PA and controlled asthma in men (Table 2: Crude OR (95%CI): 1.87 (1.06-3.28), 1.88 (1.07-3.30) and 1.95 (1.10-3.46), respectively). In women and in both genders combined, vigorous PA seemed to be associated with uncontrolled asthma (Crude OR (95%CI): 1.97 (1.14-3.39) and 1.50 (1.02-2.22), respectively).

**Table 2:** Crude odds ratios predicting controlled and uncontrolled asthma as compared to no asthma

	**Men (n = 371)**				**Women (n = 438)**				**Total (n = 809)**			
	**Controlled asthma (n = 59)**		**Uncontrolled asthma (n = 12)**		**Controlled asthma (n = 66)**		**Uncontrolled asthma (n = 66)**		**Controlled asthma (n = 125)**		**Uncontrolled asthma (n = 78)**	
	**OR**	**(95% CI)**	**OR**	**(95% CI)**	**OR**	**(95% CI)**	**OR**	**(95% CI)**	**OR**	**(95% CI)**	**OR**	**(95% CI)**
**Daily sitting time**												
≤ median^a^	1.00	(Ref)	1.00	(Ref)	1.00	(Ref)	1.00	(Ref)	1.00	(Ref)	1.00	(Ref)
> median	**1.87**	(1.06-3.28)*	0.60	(0.16-2.27)	0.95	(0.56-1.63)	1.08	(0.63-1.84)	1.32	(0.90-1.94)	1.14	(0.71-1.83)
**VPA MET-min/week**												
≤ median^a^	1.00	(Ref)	1.00	(Ref)	1.00	(Ref)	1.00	(Ref)	1.00	(Ref)	1.00	(Ref)
> median	**1.88**	(1.07-3.30)*	1.38	(0.44-4.38)	1.26	(0.72-2.22)	**1.97**	(1.14-3.39)*	**1.50**	(1.02-2.22)*	1.54	(0.96-2.47)
**MPA MET-min/week**												
≤ median^a^	1.00	(Ref)	1.00	(Ref)	1.00	(Ref)	1.00	(Ref)	1.00	(Ref)	1.00	(Ref)
> median	**1.95**	(1.10-3.46)*	1.16	(0.37-3.67)	0.92	(0.54-1.57)	1.25	(0.73-2.13)	1.31	(0.89-1.93)	1.28	(0.80-2.05)
**Walking MET-min/week**												
≤ median^a^	1.00	(Ref)	1.00	(Ref)	1.00	(Ref)	1.00	(Ref)	1.00	(Ref)	1.00	(Ref)
> median	1.27	(0.72-2.22)	1.50	(0.47-4.82)	0.87	(0.51-1.49)	0.82	(0.48-1.40)	1.04	(0.71-1.53)	0.93	(0.58-1.49)
**Total MET-min/week**												
≤ median^a^	1.00	(Ref)	1.00	(Ref)	1.00	(Ref)	1.00	(Ref)	1.00	(Ref)	1.00	(Ref)
> median	1.30	(0.74-2.28)	1.10	(0.35-3.48)	1.07	(0.63-1.82)	1.45	(0.85-2.48)	1.17	(0.80-1.72)	1.40	(0.87-2.25)
**Overall PA level**												
Low^a^	1.00	(Ref)	1.00	(Ref)	1.00	(Ref)	1.00	(Ref)	1.00	(Ref)	1.00	(Ref)
Moderate	1.06	(0.54-2.08)	1.33	(0.31-5.72)	0.70	(0.36-1.35)	0.99	(0.50-1.96)	0.86	(0.54-1.37)	1.07	(0.58-1.96)
HEPA	0.91	(0.44-1.86)	1.28	(0.28-5.87)	0.80	(0.42-1.53)	1.17	(0.59-2.29)	0.85	(0.53-1.38)	1.28	(0.70-2.33)
**2007 PA recommendation**												
No^a^	1.00	(Ref)	1.00	(Ref)	1.00	(Ref)	1.00	(Ref)	1.00	(Ref)	1.00	(Ref)
Yes	1.37	(0.78-2.40)	1.62	(0.50-5.22)	1.10	(0.65-1.87)	1.24	(0.73-2.12)	1.23	(0.88-1.80)	1.37	(0.85-2.21)

a: reference category; * p < 0.05; PA: physical activity; MPA: moderate PA; VPA: vigorous PA; Total PA: walking, moderate and vigorous PA; HEPA: health-enhancing PA.

After adjustments for confounders, moderate PA remained a predictor of controlled asthma in men (Table 3: Adjusted OR (95%CI) 1.84 (1.02-3.30)), while vigorous PA doubled the risk of uncontrolled asthma in women (Adjusted OR (95% CI) 1.94 (1.13-3.35)).

**Table 3:** Adjusted odds ratios predicting controlled and uncontrolled asthma as compared to no asthma

	**Men; controlled asthma (n = 59)**		**Women; uncontrolled asthma (n = 66)**	
	**Adjusted OR**^**b**^**(95% CI)**		**Adjusted OR**^**c**^**(95% CI)**	
**Daily sitting time**				
≤ median^a^	1.00	(Ref)	1.00	(Ref)
> median	1.47	(0.81-2.65)	1.04	(0.61-1.79)
**VPA MET-min/week**				
≤ median^a^	1.00	(Ref)	1.00	(Ref)
> median	1.52	(0.85-2.74)	**1.94**	(1.13-3.35)*
**MPA MET-min/week**				
≤ median^a^	1.00	(Ref)	1.00	(Ref)
> median	**1.84**	(1.02-3.30)*	1.27	(0.75-2.18)
**Total MET-min/week**				
≤ median^a^	1.00	(Ref)	1.00	(Ref)
> median	1.17	(0.66-2.09)	1.46	(0.85-2.49)

a: reference category; b: Adjusted for age and BMI; c: Adjusted for age; * p < 0.05; PA: physical activity; MPA: moderate PA; VPA: vigorous PA, Total PA: walking, moderate and vigorous PA; HEPA: health-enhancing PA.

## Discussion

### Comparison of physical activity levels

This observational study shows that overall physical activity levels of asthmatics are not significantly different from those of non-asthmatics. Asthmatics thus do not seem to have a more sedentary lifestyle than healthy individuals, which extends the findings of earlier studies [5,9,19]. In fact, more vigorous PA was found among controlled (men) and uncontrolled asthmatics (women) than among healthy respondents. Still, PA should be encouraged among Portuguese adults, as only half of them reached the PA recommendations and as adequate PA has numerous health benefits beyond asthma control [4].

Women who did vigorous PA were more likely to have uncontrolled asthma. This could point at vigorous PA triggering EIB. In men, controlled asthmatics more frequently did moderate PA, while vigorous PA was not linked with asthma status. Regular moderate activities thus seem protective against asthma symptoms PA [20,21], while vigorous PA seems not advisable, but these observations should be confirmed by other studies, due to a lack of power.

In literature, regular PA has been associated with decreased asthma severity and incidence and improved quality of life, while low activity levels have been associated with higher asthma rates [1,2,22,23]. Still, reverse causation cannot be excluded: asthma could lead to activity restrictions.

Next to a true absence of association between overall PA and asthma status, asthma under-diagnosis in case of low PA could also explain this finding [24]. Also, asthma may cause patients to adopt a healthier lifestyle, including more PA, thereby moderating observed associations [25].

### Strengths and limitations

This study has various strengths and limitations. First, our sample was population-based but not a random sample of Portuguese residents. Since our sample size was smaller than required, power might have been too low to detect some effects. PA levels and asthma status could for example be linked consistently in both males and females, though not observed due to small groups. The low number of male asthmatics with uncontrolled disease may be the reason for the lack of association with vigorous PA in men, in contrast to the association observed in women. The low response rate might have caused selection bias. Health conscious subjects might for example have been more likely to participate. Moreover, due to the cross-sectional nature of this study, no causal relationships can be implied, while we did not account for specific medication intake, asthma duration, etc. Also self-report may influence our results. Asthmatics might for example recall more PA due to exercise related symptoms. Still, asthma self-report is used widely and has a good specificity [26].

To our knowledge, this study is the first to compare PA levels of adult asthmatics with controlled and uncontrolled disease to those of healthy individuals. Another strength is the use of IPAQ, a standardized questionnaire to evaluate PA in populations worldwide with reasonable validity and reliability [27]. The IPAQ assesses moderate and vigorous PA and walking across four activity domains (leisure, occupation, transportation and household) and addresses time spent sitting on an ordinary day as well [27,28]. Moreover, the use of median values and data truncation minimizes misclassification of activity level [29].

## Conclusions

Portuguese adult asthmatics, independently of asthma control, do not seem to have a more sedentary lifestyle than healthy Portuguese residents. Nevertheless, PA should be encouraged, as only about half of all respondents reached activity recommendations. In addition, moderate activities seem protective against asthma symptoms, whereas vigorous activities could provoke them.

Asthma status in our population was more strongly associated with specific PA levels than with overall PA and associations depend on asthma control.

Longitudinal studies on PA in asthmatics and further intervention trials assessing the effect of PA on adult asthma are highly required. Next to elucidating cause-effect relationships, these studies should attempt to measure PA and asthma objectively and to distinguish between controlled and uncontrolled asthma and various PA dimensions.

## Abbreviations

ACSM: American College of Sports Medicine;AHA: American Heart Association;BMI: Body mass index;CATI: Computer assisted telephone interview;EIB: Exercise-induced bronchoconstriction;HEPA: Health-enhancing physical activity;IQR: Interquartile range;MET: Metabolic equivalent;MPA: Moderate physical activity;PA: Physical activity;PAQ: International Physical Activity Questionnaire;SES: Socioeconomic status;VPA: Vigorous physical activity;WPA: Walking physical activity

## Competing interests

All authors declare no competing interests.

## Authors’ contributions

VA performed the statistical analysis and drafted the manuscript. MA participated in the design of the study and helped to draft the manuscript. SSA, SR, MP and FJ participated in the design of the study and statistical analysis and helped to draft the manuscript. BR helped with statistical analysis. All authors read and approved the final manuscript.
